# Association of Apolipoprotein E ɛ4 Allele With Clinical and Multimodal Biomarker Changes of Alzheimer Disease in Adults With Down Syndrome

**DOI:** 10.1001/jamaneurol.2021.1893

**Published:** 2021-07-06

**Authors:** Alexandre Bejanin, Maria Florencia Iulita, Eduard Vilaplana, Maria Carmona-Iragui, Bessy Benejam, Laura Videla, Isabel Barroeta, Susana Fernandez, Miren Altuna, Jordi Pegueroles, Victor Montal, Silvia Valldeneu, Sandra Giménez, Sofía González-Ortiz, Laia Muñoz, Concepción Padilla, Mateus Rozalem Aranha, Teresa Estellés, Ignacio Illán-Gala, Olivia Belbin, Valle Camacho, Liam Reese Wilson, Tiina Annus, Ricardo S. Osorio, Sebastián Videla, Sylvain Lehmann, Anthony J. Holland, Henrik Zetterberg, Kaj Blennow, Daniel Alcolea, Jordi Clarimon, Shahid H. Zaman, Rafael Blesa, Alberto Lleó, Juan Fortea

**Affiliations:** 1 Sant Pau Memory Unit, Department of Neurology, Hospital de la Santa Creu i Sant Pau, Biomedical Research Institute Sant Pau, Universitat Autònoma de Barcelona, Barcelona, Spain; 2 Center of Biomedical Investigation Network for Neurodegenerative Diseases (CIBERNED), Madrid, Spain; 3 Barcelona Down Medical Center, Fundació Catalana Síndrome de Down, Barcelona, Spain; 4 Multidisciplinary Sleep Unit, Respiratory Department, Hospital de la Santa Creu i Sant Pau, Barcelona, Spain; 5 Hospital del Mar, Barcelona, Spain; 6 Nuclear Medicine Department, Hospital de la Santa Creu i Sant Pau, Barcelona, Spain; 7 Cambridge Intellectual and Developmental Disabilities Research Group, Department of Psychiatry, University of Cambridge, Douglas House, Cambridge, United Kingdom; 8 Healthy Brain Aging and Sleep Center, Department of Psychiatry, New York University Grossman School of Medicine, New York; 9 Clinical Research Support Unit, Bellvitge Biomedical Research Institute Department of Clinical Pharmacology, University of Barcelona, Barcelona, Spain; 10 Le Laboratoire de Biochimie et Protéomique Clinique, Université Montpellier, Centre Hospitalier Universitaire Montpellier, Institut National de la Santé et de la Recherche Médicale, Montpellier, France; 11 Department of Psychiatry and Neurochemistry, Institute of Neuroscience and Physiology, the Sahlgrenska Academy at the University of Gothenburg, Mönldal, Sweden; 12 Clinical Neurochemistry Laboratory, Sahlgrenska University Hospital, Mölndal, Sweden; 13 UK Dementia Research Institute at University College London (UCL), London, United Kingdom; 14 Department of Neurodegenerative Disease, UCL Queen Square Institute of Neurology, London, United Kingdom; 15 Cambridgeshire and Peterborough National Health Service (NHS) Foundation Trust, Fulbourn Hospital, Elizabeth House, Cambridge, United Kingdom

## Abstract

**Importance:**

Alzheimer disease (AD) is the leading cause of death in individuals with Down syndrome (DS). Previous studies have suggested that the *APOE* ɛ4 allele plays a role in the risk and age at onset of dementia in DS; however, data on in vivo biomarkers remain scarce.

**Objective:**

To investigate the association of the *APOE* ɛ4 allele with clinical and multimodal biomarkers of AD in adults with DS.

**Design, Setting, and Participants:**

This dual-center cohort study recruited adults with DS in Barcelona, Spain, and in Cambridge, UK, between June 1, 2009, and February 28, 2020. Included individuals had been genotyped for *APOE* and had at least 1 clinical or AD biomarker measurement; 2 individuals were excluded because of the absence of trisomy 21. Participants were either *APOE* ɛ4 allele carriers or noncarriers.

**Main Outcomes and Measures:**

Participants underwent a neurological and neuropsychological assessment. A subset of participants had biomarker measurements: Aβ1-42, Aβ1-40, phosphorylated tau 181 (pTau181) and neurofilament light chain (NfL) in cerebrospinal fluid (CSF), pTau181, and NfL in plasma; amyloid positron emission tomography (PET); fluorine 18–labeled-fluorodeoxyglucose PET; and/or magnetic resonance imaging. Age at symptom onset was compared between *APOE* ɛ4 allele carriers and noncarriers, and within-group local regression models were used to compare the association of biomarkers with age. Voxelwise analyses were performed to assess topographical differences in gray matter metabolism and volume.

**Results:**

Of the 464 adults with DS included in the study, 97 (20.9%) were *APOE* ɛ4 allele carriers and 367 (79.1%) were noncarriers. No differences between the 2 groups were found by age (median [interquartile range], 45.9 [36.4-50.2] years vs 43.7 [34.9-50.2] years; *P* = .56) or sex (51 male carriers [52.6%] vs 199 male noncarriers [54.2%]). *APOE* ɛ4 allele carriers compared with noncarriers presented with AD symptoms at a younger age (mean [SD] age, 50.7 [4.4] years vs 52.7 [5.8] years; *P* = .02) and showed earlier cognitive decline. Locally estimated scatterplot smoothing curves further showed between-group differences in biomarker trajectories with age as reflected by nonoverlapping CIs. Specifically, carriers showed lower levels of the CSF Aβ1-42 to Aβ1-40 ratio until age 40 years, earlier increases in amyloid PET and plasma pTau181, and earlier loss of cortical metabolism and hippocampal volume. No differences were found in NfL biomarkers or CSF total tau and pTau181. Voxelwise analyses showed lower metabolism in subcortical and parieto-occipital structures and lower medial temporal volume in *APOE* ɛ4 allele carriers.

**Conclusions and Relevance:**

In this study, the *APOE* ɛ4 allele was associated with earlier clinical and biomarker changes of AD in DS. These results provide insights into the mechanisms by which *APOE* increases the risk of AD, emphasizing the importance of *APOE* genotype for future clinical trials in DS.

## Key Points

**Question:**

What is the association of the apolipoprotein E (*APOE*) ɛ4 allele with Alzheimer disease–related clinical and biomarker changes in Down syndrome?

**Findings:**

In this cohort study of 464 adults with Down syndrome, carriers of the *APOE* ɛ4 allele showed both earlier clinical symptoms of Alzheimer disease and earlier changes in amyloid (cerebrospinal fluid Aβ1-42/1-40 and amyloid positron emission tomography), tau (plasma phosphorylated tau 181), and neurodegeneration (cerebral glucose hypometabolism and hippocampal atrophy) biomarkers. The *APOE* ɛ4 allele also altered the topography of neurodegeneration.

**Meaning:**

Results of this study suggest that the *APOE* ɛ4 allele can modulate both the clinical expression and the biomarkers of Alzheimer disease in a genetic form of the disease, such as in Down syndrome.

## Teaser

This cohort study examines Alzheimer disease symptoms and age at onset; changes in amyloid, tau, and neurodegeneration biomarkers; and topography of brain changes in adults with Down syndrome.

## Introduction

Individuals with Down syndrome (DS) constitute a population at ultrahigh risk of developing Alzheimer disease (AD) because of trisomy of chromosome 21, which harbors the *APP* (amyloid precursor protein; OMIM 104760) gene. Recent estimates indicate a lifetime dementia risk of more than 90% and identify Alzheimer dementia as the leading cause of death in this population.^1^ This elevated risk has led to the conceptualization of DS as a genetically determined form of AD that is similar to autosomal dominant forms. This idea is further supported by a recent study that showed that the pattern of AD biomarker changes follows a similar temporal profile in DS as in autosomal dominant forms, with a long preclinical phase and pathophysiological processes that are qualitatively comparable to sporadic AD.^2^

The apolipoprotein E (*APOE*; OMIM 107741) ε4 allele is the most established genetic risk factor for sporadic AD and has been consistently associated with earlier AD symptoms^3,4^ and pathology^5,6,7^ in the general population. A similar disease-accelerating feature might exist in DS given that studies in this population have reported that ɛ4 allele carriers show an earlier onset of clinical symptoms^8,9,10,11^ and greater amyloid burden^12^ than noncarriers. However, little is known about the association of the *APOE* ɛ4 allele with the evolution of AD biomarkers.

Using biochemical and neuroimaging measures for all 3 categories of the ATN system^13^ (ie, amyloid, tau, and neurodegeneration), we conducted this cohort study to investigate the association of the *APOE* ɛ4 allele with clinical and multimodal biomarker changes of AD in adults with DS. We also examined the association of the *APOE* ɛ4 allele with the topography of structural and functional brain changes.

## Methods

This cohort study was approved by the Clinical Research Ethics Committee at Hospital Sant Pau and the University of Cambridge Research Ethics Committee and by the Administration of Radioactive Substances Advisory Committee. In Spain, all study participants or their legally authorized representatives gave written informed consent before study enrollment. In the United Kingdom, written consent was obtained from all adults with DS who had the capacity to consent. For participants in England and Wales who lacked the capacity to consent, the procedures in the Mental Capacity Act of 2005 were followed.

Between June 1, 2009, and February 28, 2020, we recruited adults with DS. In Barcelona, Spain, adults with DS were recruited from a population-based health plan that was developed for the screening of AD from which the Down Alzheimer Barcelona Neuroimaging Initiative cohort was formed.^2,18^ In Cambridge, UK, participants were selected from a convenience sample that was recruited from services for people with intellectual disabilities in England and Scotland.^14^

The study included adults with DS who were screened for the *APOE* genotype and underwent a comprehensive clinical evaluation. Most, but not all, of these individuals had at least 1 biochemical or imaging AD biomarker assessment. Genetic screening confirmed complete trisomy 21 in 98.3% of the individuals who were assessed; 2 individuals were excluded because of the absence of trisomy 21.

### Clinical and Neuropsychological Assessment

The *Diagnostic and Statistical Manual of Mental Disorders, Fifth Edition* was used to stratify the level of intellectual disability as mild, moderate, or severe or profound. Participants were further classified as having asymptomatic, prodromal, or AD dementia in a consensus meeting between the neurologist or psychiatrist and neuropsychologist (eMethods in the Supplement).

Global cognition was assessed using the Cambridge Cognitive Examination for Older Adults with Down Syndrome (CAMCOG-DS),^15^ and episodic memory was evaluated with the modified Cued Recall Test (mCRT).^16^ The mCRT was adapted for people with intellectual disabilities and consisted of 3 trials of free and cued recall performed both immediately and approximately 15 to 20 minutes after the learning phase. Free and cued performances were summed to obtain total scores of immediate and delayed recall. To account for the association of intellectual disability with cognitive performances, we excluded severe or profound cases to prevent floor effects^17^ and *z*-transformed the cognitive performances in the mild and moderate intellectual disability groups separately.

### *APOE* Genotyping, Fluid Biomarkers, and Neuroimaging

DNA was extracted from peripheral blood by technicians who were blinded to clinical and biomarker data, and *APOE* genotyping was determined by polymerase chain reaction amplification.^18^ Participants were dichotomized according to the presence of at least 1 ɛ4 allele.

Cerebrospinal fluid (CSF) and blood samples were acquired, as previously described.^18,19^ Plasma levels of phosphorylated tau 181 (pTau181) and neurofilament light chain (NfL) were measured using single molecule array technology (Simoa; Quanterix). The pTau181 analyses were carried out at the University of Gothenburg (Sweden) using a validated assay.^20^ The analysis of NfL was performed at the Centre Hospitalier Universitaire de Montpellier (France) and Hospital Sant Pau (Spain).^2,18^ The CSF levels of amyloid-β peptide 1-40 (Aβ1-40), Aβ1-42, pTau181, and total tau were quantified using a fully automated platform (Lumipulse; Fujirebio), following the published protocol.^21^ The CSF NfL levels were measured with enzyme-linked immunosorbent assay (NF-Light Assay; UmanDiagnostics) according to the manufacturer recommendations.

A subset of participants underwent 3-T magnetic resonance imaging (MRI; n = 175), fluorine 18–labeled (^18^F) fluorodeoxyglucose (FDG) positron emission tomography (PET; n = 132), and/or amyloid PET (n = 75). The Computational Anatomy Toolbox (CAT12; Christian Gaser and Robert Dahnke) for the SPM12 software (Wellcome Centre for Human Neuroimaging, University College London, Queen Square Institute of Neurology) was used to preprocess the structural, 3-dimensional, T1-weighted MRI and extract the hippocampal and total intracranial volumes. The ^18^F-FDG PET images were intensity scaled by the pons-vermis region and spatially normalized using the SPM12 software. The standardized uptake value ratios were extracted from the region of interest in Landau et al.^23^ Both the ^18^F-FDG PET and the segmented and modulated gray matter maps were smoothed using an 8-mm full-width at half-maximum Gaussian kernel for voxelwise analyses. The amyloid PET data were collected using ^18^F-florbetapir in Barcelona and carbon 11–labeled Pittsburg compound B in Cambridge. Images were spatially normalized using the MRI transformations computed with Advanced Normalization Tools^24^ and scaled using the whole cerebellum as the reference region.^2,25,26^ The mean cortical standardized uptake value ratio^22^ was then transformed into centiloid units according to standard procedures.^27^

### Statistical Analysis

All statistical analyses were performed with R software, version 4.0.4 (R Foundation for Statistical Computing). Differences in baseline characteristics, percentage of symptomatic cases across age intervals, and age at diagnosis were analyzed using χ^2^ tests (or Fisher exact tests, when appropriate) for categorical data, and Mann-Whitney or 2-sample, unpaired, 2-tailed *t* tests were used for continuous variables. Survival analysis with a log-rank test was also conducted to assess between-group differences in the age at the first diagnosis of symptomatic AD (which combines prodromal and dementia cases). The threshold for significance was set at *P* < .05.

To compare the age-associated changes in cognition and biomarkers between *APOE* ɛ4 allele carriers and noncarriers, we fitted a first-order locally estimated scatterplot smoothing curve, with a tricubic weight function and a span parameter of 0.75, in each group independently. Given that no ɛ4 allele carriers were older than 60 years, the curves did not include noncarriers older than carriers for a more uniform comparison. The exact age at which the intervals diverge depends on the intrinsic limitations of the study, such as the nature of the variable, the sensitivity of the assay, the slope of the association, and, in the present case, the uneven sample sizes for the different measurements. Therefore, we defined cognitive or biomarker change as the age at which the groups’ curves appeared to start diverging visually and provided the age range at which the 95% CIs between groups did not overlap. A convenience sample of cognitively unimpaired euploid participants (n = 158; eTable 1 in the Supplement) from the Sant Pau Initiative on Neurodegeneration cohort^21^ was included in the analyses as a visual reference of the biomarker changes occurring in individuals without trisomy 21. In addition to the locally estimated scatterplot smoothing analyses, we conducted between-group comparisons for each biomarker and each decade using Mann-Whitney tests (eFigures 2-4 in the Supplement).

We used voxelwise linear models to compare the topography of gray matter metabolism and volume in ɛ4 allele carriers vs noncarriers. Analyses were performed in a mask that excluded non–gray matter voxels, and the statistical models were corrected for age and sex as well as for total intracranial volume and recruitment center for the models that included volume. Voxelwise results are presented at an uncorrected threshold of *P* < .001 (cluster size k >100 mm^3^).

## Results

Of the 464 adults with DS included, 97 (20.9%) were *APOE* ɛ4 allele carriers (ɛ2/4: n = 7; ɛ3/4: n = 86; and ɛ4/4: n = 4) and 367 (79.1%) were noncarriers. The 2 groups did not differ significantly in age (median [interquartile range (IQR)], 45.9 [36.4-50.2] years vs 43.7 [34.9-50.2] years; *P* = .56), sex distribution (male participants: 51 [52.6%] vs 199 [54.2%]; female participants: 46 [47.4%] vs 168 [45.8%]; *P* = .86), level of intellectual disability (mild: 23 [23.7%] vs 82 [22.5%]; moderate: 50 [51.6%] vs 190 [52.2%]; and severe or profound: 24 [24.7%] vs 92 [25.3%]; *P* = .97), or the most common health conditions observed in DS (eg, hypothyroidism: 29 [44.6% of those evaluated] vs 124 [51.2% of those evaluated]; *P* = .42) (Table; eTable 2 in the Supplement has details on the subsamples for each biomarker). Similar to the general population, the ɛ3 allele was the most prevalent, followed by ɛ4 and ɛ2 alleles. When comparing recruiting sites, we found no demographic, genetic, or clinical differences between the Barcelona and Cambridge cohorts except for intellectual disability (eTable 3 in the Supplement).

**Table.** Study Participants^a^

Variable	*APOE* ɛ4 allele noncarrier	*APOE* ɛ4 allele carrier	*P* value
All participants (n = 464), No. (%)	367 (79.1)	97 (20.9)	NA
Age, median (IQR), y	43.7 (34.9-50.2)	45.9 (36.4-50.2)	.56
Sex, No. (%)			
Female	168 (45.8)	46 (47.4)	.86
Male	199 (54.2)	51 (52.6)	
Level of intellectual disability, No. (%)^b^			
Mild	82 (22.5)	23 (23.7)	.97
Moderate	190 (52.2)	50 (51.6)	
Severe or profound	92 (25.3)	24 (24.7)	
Diagnostic group, No. (%)^b^			
Asymptomatic AD	256 (70.1)	60 (63.2)	.24
Symptomatic AD	109 (29.9)	35 (36.8)	
*APOE* alleles, No.			NA
ɛ2/ɛ2	1	0	
ɛ2/ɛ3	47	0	
ɛ2/ɛ4	0	7	
ɛ3/ɛ3	319	0	
ɛ3/ɛ4	0	86	
ɛ4/ɛ4	0	4	
Medical conditions, No. (%)^b^			
Hypothyroidism (n = 307)	124 (51.2)	29 (44.6)	.42
Epilepsy (n = 289)	19 (8.3)	8 (13.3)	.35
Sleep apnea (n = 297)	27 (11.4)	11 (18.3)	.22
Depression (n = 330)	34 (13.1)	13 (18.3)	.36
Cognition, median (IQR)^c^			
CAMCOG-DS score (n = 301)	74.0 (59.0-85.0)	73.5 (63.0-83.0)	.68
mCRT immediate recall (n = 263)	35.0 (33.0-36.0)	35.0 (31.0-36.0)	.14
mCRT delayed recall (n = 262)	12.0 (10.0-12.0)	12.0 (9.0-12.0)	.30
Fluid biomarkers, median (IQR)			
CSF Aβ1-42/1-40 (n = 156)	0.1 (<0.1-0.1)	0.1 (<0.1-0.1)	.08
CSF NfL (n = 139)	493.7 (305.6-791.9)	663.9 (372.5-880.0)	.47
CSF pTau181 (n = 158)	44.2 (25.8-122.3)	71.8 (32.0-119.7)	.29
CSF total tau (n = 158)	398.5 (247.8-755.0)	516.0 (236.0-749.0)	.82
Plasma NfL (n = 354)	9.6 (5.3-15.3)	10.7 (6.3-16.8)	.11
Plasma pTau181 (n = 354)	12.5 (8.6-22.2)	17.8 (11.4-25.8)	.007
Imaging biomarkers, median (IQR)			
Centiloid amyloid PET (n = 75)^d^	7.6 (0.3-33.8)	17.4 (5.0-67.7)	.08
^18^F-FDG PET SUVR (n = 132)	1.3 (1.1-1.4)	1.2 (0.9-1.4)	.44
Bilateral hippocampal volume (n = 175)	6.8 (6.2-7.4)	6.5 (5.1-7.4)	.20
Bilateral hippocampal volume per TIV (n = 175)	0.0059 (0.0053-0.0062)	0.0057 (0.0047-0.0062)	.16

Abbreviations: Aβ1-40, amyloid-β peptide 1-40; Aβ1-42, amyloid-β peptide 1-42; AD, Alzheimer disease; *APOE*, apolipoprotein E; CAMCOG-DS, Cambridge Cognitive Examination for Older Adults with Down Syndrome; CSF, cerebrospinal fluid; ^18^F FDG, fluorine 18–labeled fluorodeoxyglucose; IQR, interquartile range; mCRT, modified Cued Recall Test; NA, not applicable; NfL, neurofilament light chain; PET, positron emission tomography; pTau181, phosphorylated tau 181; SUVR, standardized uptake value ratio; TIV, total intracranial volume.

^a^ Unless otherwise indicated, values were number (%) or median (IQR). All fluid biomarker concentration units, except for the CSF Aβ1-42 to Aβ1-40 ratio, were picograms per milliliter. *P* values refer to analyses of χ^2^ tests for categorical variables and Mann-Whitney tests for continuous variables.

^b^ Percentages for intellectual disability, diagnostic group, and medical conditions were calculated according to the total of patients with available data in each group.

^c^ Cognition included cognitive performances only for individuals with DS who had mild or moderate intellectual disability.

^d^ Forty-five participants had amyloid PET with fluorine 18–labeled florbetapir, and 30 participants had amyloid PET with carbon 11–labeled Pittsburgh compound B.

### Clinical and Neuropsychological Findings

**Figure 1.**
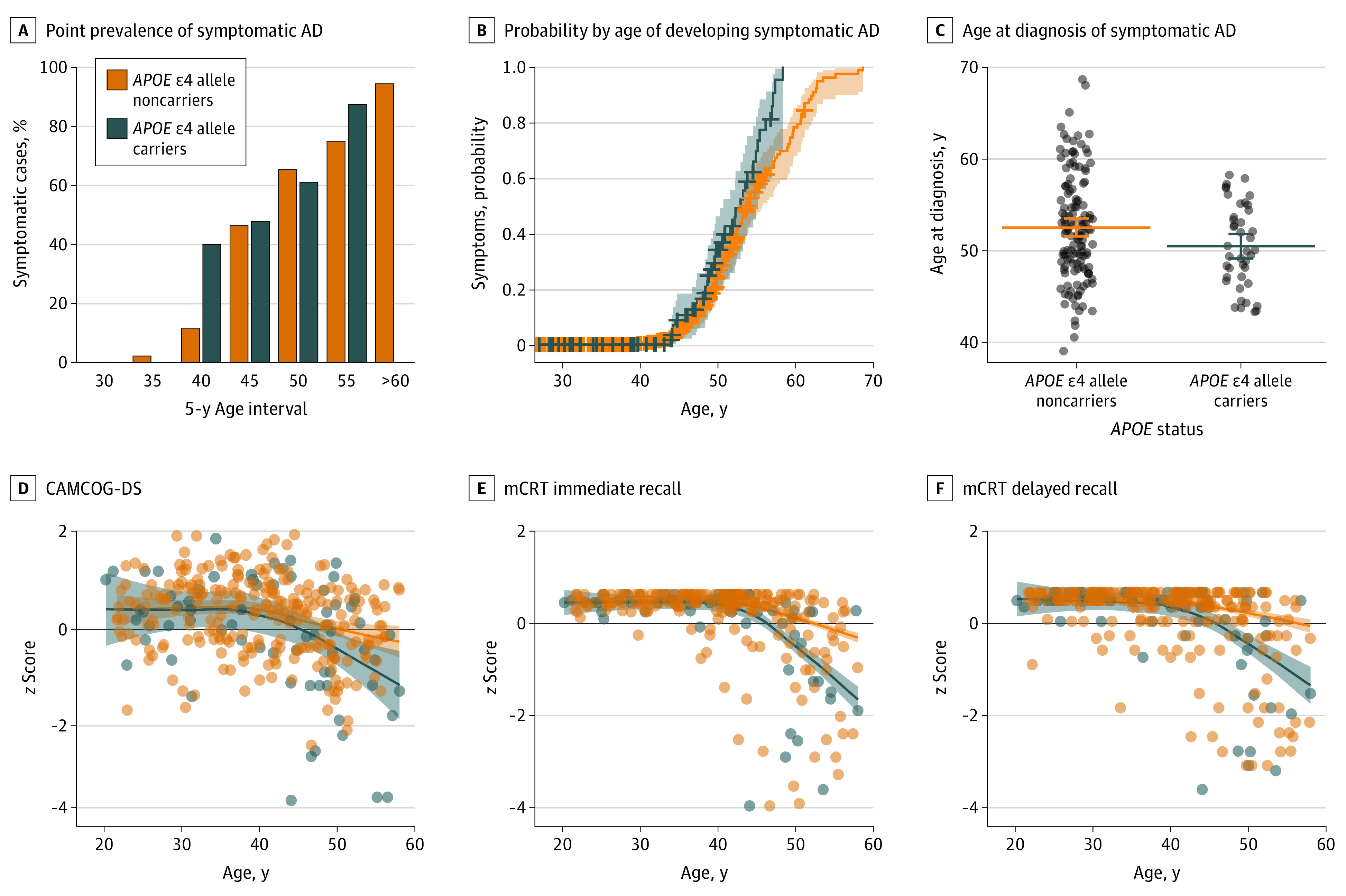
Association of Apolipoprotein E (*APOE*) ɛ4 Allele With Clinical Diagnosis and Cognitive Performance in Adults With Down Syndrome (DS) The horizontal lines in panel C represent the mean values, and the error bars represent the nonparametric bootstrapped 95% CIs. Bands in panels D to F represent the 95% CIs. AD indicates Alzheimer disease; CAMCOG-DS, Cambridge Cognitive Examination for Older Adults with Down Syndrome; and mCRT, modified Cued Recall Test.

The overall proportion of individuals with DS with a diagnosis of symptomatic AD was similar between *APOE* ɛ4 allele carriers and noncarriers (36.8% vs 29.9%; χ^2^_1,460_ = 1.4; *P* = .24). However, an age-stratified analysis by 5-year intervals revealed 2 key differences (Figure 1A). First, in the 40 to 45 years of age range, the prevalence of symptomatic AD was 40% in carriers and 12% in noncarriers (χ^2^_1,75_ = 4.9; *P* = .03). Second, in contrast to the noncarrier group in which 18 individuals were older than 60 years, we found no individuals carrying the ɛ4 allele who were older than 60 years, even when examining data from follow-up visits.

Next, we used baseline and follow-up data to examine the age at which individuals with DS had been diagnosed with symptomatic AD. The survival curve showed a significant difference in the distributions of the 2 groups (log-rank test, *P* = .01), with the carrier group showing a greater probability of being diagnosed with symptomatic AD at an earlier age (Figure 1B). The between-group comparison further confirmed this earlier age at diagnosis in carriers compared with noncarriers (mean [SD] age, 50.7 [4.4] years vs 52.7 [5.8] years; *P* = .02) (Figure 1C).

We also evaluated the performance on the CAMCOG-DS and mCRT tests (immediate and delayed recall) expressed as *z* scores and as a function of age (eFigures 1 and 2 in the Supplement have raw data and analyses in participants with mild and moderate levels of intellectual disability). All participants with DS showed decreased CAMCOG-DS *z* scores with age regardless of *APOE* genotype. Visually, the change seemed earlier and steeper in *APOE* ɛ4 allele carriers compared with noncarriers starting from age 40 years, but the CIs did not diverge (Figure 1D). Carriers also showed a decrease in both the immediate and delayed recall scores of the mCRT at an earlier age compared with noncarriers (Figure 1E and F). The difference between the 2 groups was evident from age 40 years (nonoverlapping CIs from age 44 years). These age-associated differences were not reflected by significant group differences, when the mCRT scores were compared between the whole group of carriers and noncarriers (median (IQR) immediate recall: 35.0 [31.0-36.0] vs 35.0 [33.0-36.0]; *P* = .14; median (IQR) delayed recall: 12.0 [9.0-12.0] vs 12.0 [10.0-12.0]; *P* = .30) (Table).

### Amyloid and Tau Pathology Biomarkers

**Figure 2.**
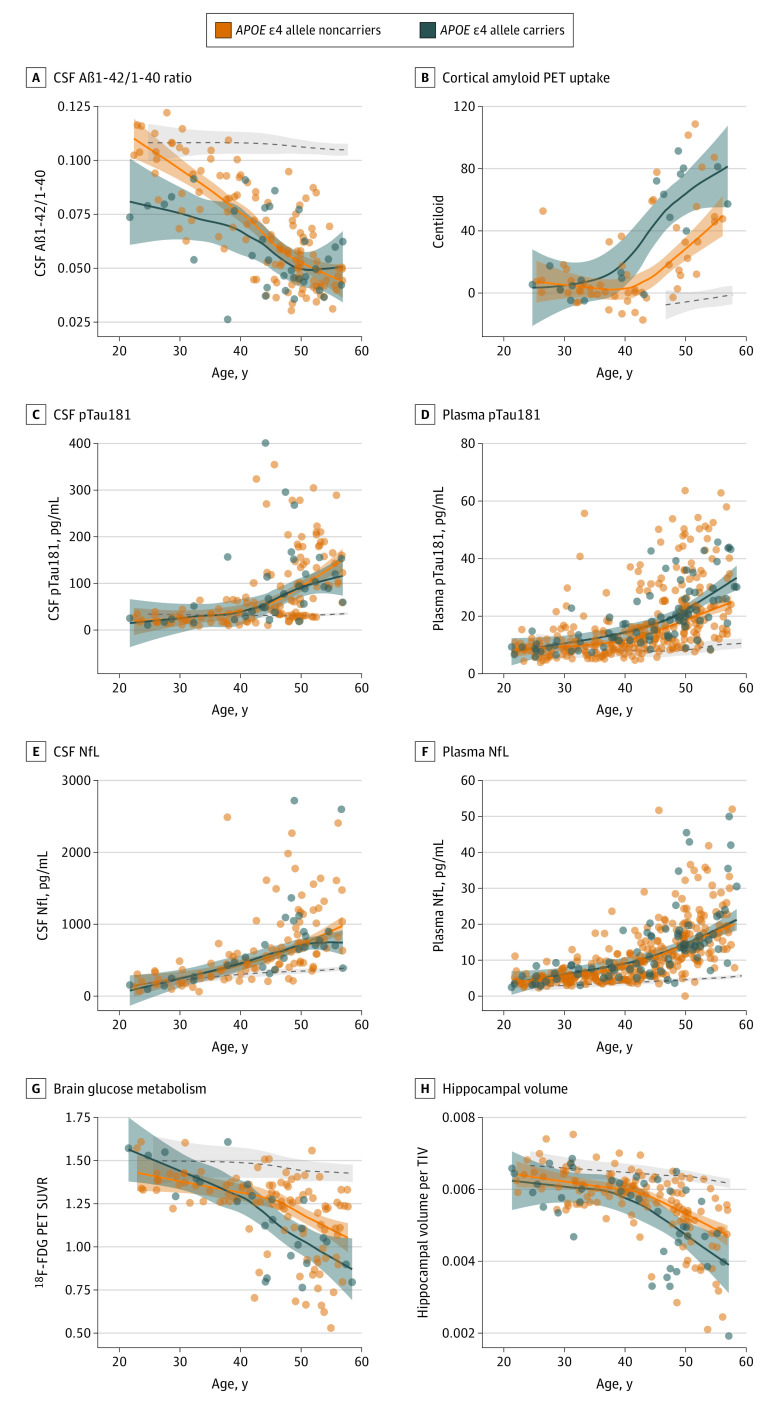
Association of Apolipoprotein E (*APOE*) ɛ4 Allele With Age-Related Changes in Alzheimer Disease (AD) Biomarkers Shading represents 95% CIs, and the dashed lines represent the age-related changes in euploid individuals for visual reference. Aβ1-40 indicates amyloid-β peptide 1-40; Aβ1-42, amyloid-β peptide 1-42; CSF, cerebrospinal fluid; ^18^F-FDG, fluorine 18–labeled fluorodeoxyglucose; NfL, neurofilament light chain; PET, positron emission tomography; pTau181, phosphorylated tau 181; SUVR, standardized uptake value ratio; and TIV, total intracranial volume.

The CSF Aβ1-42 to Aβ1-40 ratio was lower in *APOE* ɛ4 allele carriers than in noncarriers among the youngest participants (20s to 30s) and overlapped with noncarriers starting from age 40 years (Figure 2A and eFigure 3A in the Supplement). This result was driven by the lower levels of CSF Aβ1-42 in carriers (eFigure 4A and D in the Supplement). Similarly, carriers showed an earlier and greater increase in cortical amyloid PET uptake than noncarriers (mid-30s vs early 40s), with nonoverlapping CIs throughout the older ages (41 to 54 years) (Figure 2B) up to when amyloid uptake seemed to plateau.

The age-associated trajectories of CSF pTau181 (Figure 2C) did not differ between ɛ4 allele carriers and noncarriers. However, when analyzing this biomarker in plasma in a larger sample, carriers showed higher levels starting from the mid-40s and with CIs not overlapping by age 50 years (Figure 2D).

### Neurodegeneration Biomarkers

No between-group differences in the levels of NfL were found in CSF or in plasma as seen by the overlapping CIs across all ages and in the analyses by decade (Figure 2E and F; eFigure 3E and F in the Supplement). The CSF total tau likewise showed no differences between groups (eFigure 4C and F in the Supplement).

By contrast, neuroimaging biomarkers revealed significant differences between *APOE* genotypes. Specifically, the ɛ4 allele carriers showed lower brain metabolism than noncarriers starting at age 40 years. This difference was sustained until the sixth decade, and the CIs of both groups diverged from age 45 to 53 years (Figure 2G and eFigure 3G in the Supplement). Similarly, carriers showed an earlier loss of hippocampal volume starting from age 40 years and with nonoverlapping CIs from age 48 to 52 years (Figure 2H and eFigure 3H in the Supplement).

### Topography of Brain Hypometabolism and Atrophy

**Figure 3.**
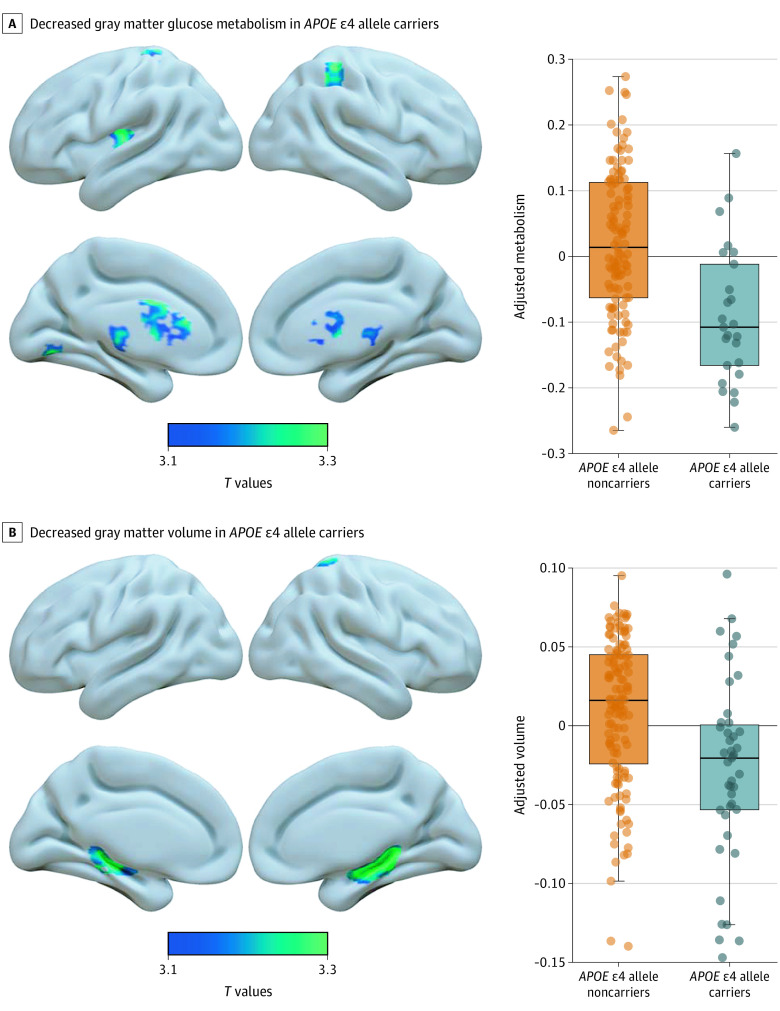
Association of Apolipoprotein E (*APOE*) ɛ4 Allele With Gray Matter Metabolism and Volume The boxplots illustrate the voxelwise results and represent the metabolism (A) and volume (B) adjusted for the covariates within the significant clusters. For each boxplot, the box represents the interquartile range, the band represents the median value, and the dots represent individual values. Results are presented in neurological convention and were generated using the Surf Ice tool (https://www.nitrc.org/projects/surfice/). eFigures 5 and 6 in the Supplement show a slice-by-slice display of the results.

The voxelwise analysis that was adjusted by age and sex revealed lower metabolism in the *APOE* ɛ4 allele carriers compared with noncarriers in subcortical structures (caudate, lentiform nucleus, and thalamus), posterior insula, medial, and lateral parietal and occipital cortices (Figure 3A and eFigure 5 in the Supplement). No brain region showed higher metabolism in carriers than in noncarriers.

In addition, carriers showed lower gray matter volume in the hippocampus bilaterally and right superior parietal cortex compared with noncarriers (Figure 3B and eFigure 6 in the Supplement). No brain region showed greater volume in carriers than in noncarriers.

## Discussion

To our knowledge, this is the first large, multimodal biomarker study to characterize the association of the *APOE* ɛ4 allele with clinical and biomarker changes of AD in DS. We found that *APOE* ɛ4 allele carriers (1) presented an earlier decline in episodic memory and were diagnosed with symptomatic AD a mean of 2 years before noncarriers, (2) exhibited earlier changes in AD biomarkers, and (3) showed differences in the topography of structural and functional brain changes. These results demonstrate that the *APOE* ɛ4 allele can modulate both the clinical expression and changes in AD biomarkers in a genetic form of the disease.

It has been well established that the *APOE* ɛ4 allele is associated with an increased risk of sporadic AD and younger age at onset.^3,4^ This association is also true in autosomal dominant forms, in which the ɛ4 allele is associated with earlier diagnoses.^28^ In line with this finding, we observed an association of the *APOE* ɛ4 allele with earlier decline in episodic memory and earlier clinical AD diagnoses in individuals with DS. This observation is in agreement with previous findings in other DS cohorts that showed greater cognitive impairment among ɛ4 allele carriers^29^ and a similar 2-year advancement in symptomatic AD diagnosis.^8,11^

We observed this earlier age at symptom onset despite no significant difference in the overall proportion of ɛ4 allele carriers between asymptomatic and symptomatic individuals. Discrepant results in DS have been reported concerning the ɛ4 allele proportions in these clinical groups, with some studies showing more carriers in the symptomatic group and others finding no differences.^10,30^ Although no overall group differences were found, the age-stratified analysis that we conducted revealed an increased prevalence of symptomatic AD in carriers aged 40 to 45 years. This result further emphasizes that age and sample composition are critical variables to interpreting results in the context of DS, wherein AD is inexorable. Note that no individuals with DS older than 60 years were found in the ɛ4 allele carrier group, a finding that is consistent with previous studies that associated the ɛ4 allele with lower life expectancy.^8,11^

The results also provided new in vivo pathophysiological data with which to interpret the clinical findings. We found that the *APOE* ε4 allele was associated with age-associated changes in the 3 categories of the ATN system (amyloid, tau, and neurodegeneration). The associations with amyloid pathology appeared to be the earliest (differences in the early 20s for CSF Aβ1-42 to Aβ1-40 ratio and in the mid-30s for amyloid PET), the greatest (highest magnitude), and the most consistent across the different biomarkers. Specifically, we found lower levels of CSF Aβ1-42 and CSF Aβ1-42 to Aβ1-40 ratio associated with the *APOE* ɛ4 allele. This finding reproduces previous observations in both sporadic AD^5,31,32,33^ and autosomal dominant AD.^34,35^ It also fits with the lower age-adjusted level of CSF Aβ1-42 reported in ɛ4 allele carriers with DS.^36^ The early decrease in CSF Aβ1-42 to Aβ1-40 ratio in carriers supports the finding that cerebral Aβ deposition occurs at a young age in DS and is consistent with the abundant Aβ42-immunoreactive diffuse plaques reported in teenagers and young adults in this population.^37^

The differences in CSF amyloid biomarkers were concordant with the earlier and higher increase in amyloid PET uptake in ɛ4 allele carriers (mid-30s) compared with noncarriers (mid-40s). As in sporadic and other genetic forms of AD,^5,32^ the *APOE* ɛ4 allele might be associated with an earlier and greater brain amyloid accumulation in DS. This idea is supported by postmortem data showing a greater burden of amyloid plaques in carriers with DS than noncarriers.^12^

Besides the association with amyloid pathology, the ɛ4 allele altered the age-related trajectory of plasma, but not CSF, pTau181. This association was noticeable around age 50 years, only a few years before AD symptom onset. Controversial results have been reported concerning the association between biochemical measures of pTau181 and the *APOE* ɛ4 allele, with some studies finding an association^38^ but others finding no association.^5,32,33,35^ In the present study, the differences in sample sizes between plasma pTau181 and CSF pTau181 measures (354 vs 158 participants) may partially account for the different results. The discrepancy may also be explained by the existence of other variables that modulate the association between the *APOE* ε4 allele and tau pathology, such as biological sex.^38,39^ Alternatively, the biochemical measurements across different biofluids may not consistently capture the pathological changes that occur in some specific brain structures, such as the higher tau pathology in the medial temporal lobe previously reported in ɛ4 allele carriers with sporadic AD.^40^ A similar explanation might account for the lack of association that we observed with biochemical markers of neurodegeneration (plasma and CSF NfL; CSF total tau), despite differences in neuroimaging biomarkers (^18^F-FDG PET and MRI).

Both neuroimaging biomarkers showed diverging trajectories between *APOE* ɛ4 allele carriers and noncarriers around age 40 years. The voxelwise analyses further indicated that the *APOE* ɛ4 allele was associated with not only accelerated onset of biomarker changes but also differences in the extent and topography of the pattern of neurodegeneration. Carriers had lower glucose metabolism in subcortical structures and several cortical areas, including the medial parietal region. Moreover, they presented with less medial temporal volume compared with noncarriers. These results mirror previous findings in sporadic AD that showed decreased parietal metabolism and hippocampal volume in carriers.^41^ In addition, we found an earlier decline in episodic memory in carriers starting at age 40 years. This observation corroborates the literature on the association of the *APOE* ɛ4 allele with episodic memory in the general population,^42^ which has also been suggested in DS.^43^ Given the crucial role of the hippocampus in episodic memory, its greater atrophy likely underlies the earlier memory deficits in carriers. Carriers also showed a lower metabolism in the striatum. This finding, which is not typically reported in sporadic AD, might reflect the greater vulnerability of the striatum to AD pathology in genetically determined forms of AD.^44,45^

Overall, this study provided evidence that the *APOE* ɛ4 allele exerts a similar association with AD pathophysiological processes in DS as in the general population. The association with amyloid pathology and the concurrent shift toward greater hippocampal atrophy and memory impairments in *APOE* ɛ4 allele carriers resemble the association of the *APOE* ɛ4 allele with the earlier clinical symptoms and pathogenesis of sporadic AD.^41^ Several molecular mechanisms have been proposed by which the ɛ4 isoform may alter AD pathology, including the impairment of amyloid clearance and the promotion of its deposition into amyloid plaques.^46^ Growing evidence also suggests that the *APOE* ε4 allele might contribute to tau aggregation that is independent of amyloid-β,^47^ and an autopsy study supports an association between truncated *APOE* forms and tau pathology in DS.^48^ Future studies that use ultrasensitive methods may explore these truncated forms in body fluids from people with DS.

Overall, this study capitalized on the largest cohort of adults with DS with clinical assessments and multimodal biomarkers to inform on the age-related association of the *APOE* ɛ4 allele with in vivo AD biomarkers, increasing the understanding of the mechanisms that link *APOE* to the acceleration of disease in genetically at-risk populations. We believe that this work is timely in the emerging landscape of preventive trials for dementia in DS given that consideration of *APOE* genotype might be important for drugs that are designed to lower amyloid burden and/or trials that use MRI as a surrogate marker of improved outcomes.

### Limitations

This study has limitations. The cross-sectional design and the relatively small sample sizes for some biomarkers (eg, amyloid PET) did not allow for an investigation of the differences between each of the *APOE* genotypes. Nevertheless, note that age can be used as a proxy for disease progression in genetically determined AD, and the results remained essentially similar when sensitivity analyses were performed in the subsample with the ɛ3 and ɛ4 polymorphisms.

## Conclusions

In this cohort study, *APOE* ɛ4 allele carriers (compared with noncarriers) showed an earlier decline in episodic memory, earlier clinical diagnosis of symptomatic AD, earlier changes in AD biomarkers, and differences in the pattern of neurodegeneration. These findings demonstrate that the *APOE* ɛ4 allele can modulate both the clinical expression and biomarkers of AD in a genetic form of the disease, such as in DS, and emphasize the importance of the *APOE* genotype for future clinical trials in DS.

## Notes

Supplement. **eMethods.** Diagnostic Procedure **eTable 1.** Demographic Data of the Euploid Controls Used as Reference **eTable 2.** Demographic Data of the Subsamples for Each Biomarker Modality **eTable 3.** Demographic, Genetic and Clinical Data for Each Recruiting Site **eFigure 1.** Effect of *APOE* ɛ4 on the Age-Related Cognitive Performance in Adults With Down Syndrome With Mild and Moderate Intellectual Disability **eFigure 2.** Effect of *APOE* ɛ4 on the Evolution of Cognitive Performance Analyzed by Decades in Adults With Down Syndrome With Mild and Moderate Intellectual Disability **eFigure 3.** Effect of *APOE* ɛ4 on the Evolution of Alzheimer Disease Biomarkers Analyzed by Decades in Adults With Down Syndrome **eFigure 4.** Effect of *APOE* ɛ4 on the CSF Levels of Aβ1-42, Aβ1-40, and Total Tau in Adults With Down Syndrome **eFigure 5.** Results of Voxel-Wise Linear Models Showing Lower Grey Matter Glucose Metabolism in *APOE* ɛ4 Carriers Compared With Non-Carriers **eFigure 6.** Results of Voxel-Wise Linear Models Showing Lower Grey Matter Volume in *APOE* ɛ4 Carriers Compared With Non-Carriers

## References

[1] Hithersay R, Startin CM, Hamburg S, . Association of dementia with mortality among adults with Down syndrome older than 35 years. JAMA Neurol. 2019;76(2):152-160. doi:10.1001/jamaneurol.2018.3616 30452522 PMC6439956

[2] Fortea J, Vilaplana E, Carmona-Iragui M, . Clinical and biomarker changes of Alzheimer’s disease in adults with Down syndrome: a cross-sectional study. Lancet. 2020;395(10242):1988-1997. doi:10.1016/S0140-6736(20)30689-9 32593336 PMC7322523

[3] Corder EH, Saunders AM, Strittmatter WJ, . Gene dose of apolipoprotein E type 4 allele and the risk of Alzheimer’s disease in late onset families. Science. 1993;261(5123):921-923. doi:10.1126/science.8346443 8346443

[4] Strittmatter WJ, Saunders AM, Schmechel D, . Apolipoprotein E: high-avidity binding to beta-amyloid and increased frequency of type 4 allele in late-onset familial Alzheimer disease. Proc Natl Acad Sci U S A. 1993;90(5):1977-1981. doi:10.1073/pnas.90.5.1977 8446617 PMC46003

[5] Morris JC, Roe CM, Xiong C, . APOE predicts amyloid-beta but not tau Alzheimer pathology in cognitively normal aging. Ann Neurol. 2010;67(1):122-131. doi:10.1002/ana.21843 20186853 PMC2830375

[6] Reiman EM, Caselli RJ, Yun LS, . Preclinical evidence of Alzheimer’s disease in persons homozygous for the epsilon 4 allele for apolipoprotein E. N Engl J Med. 1996;334(12):752-758. doi:10.1056/NEJM199603213341202 8592548

[7] Mishra S, Blazey TM, Holtzman DM, . Longitudinal brain imaging in preclinical Alzheimer disease: impact of APOE ε4 genotype. Brain. 2018;141(6):1828-1839. doi:10.1093/brain/awy103 29672664 PMC5972633

[8] Prasher VP, Sajith SG, Rees SD, . Significant effect of APOE epsilon 4 genotype on the risk of dementia in Alzheimer’s disease and mortality in persons with Down syndrome. Int J Geriatr Psychiatry. 2008;23(11):1134-1140. doi:10.1002/gps.2039 18464295 PMC2714805

[9] Schupf N, Kapell D, Lee JH, . Onset of dementia is associated with apolipoprotein E epsilon4 in Down’s syndrome. Ann Neurol. 1996;40(5):799-801. doi:10.1002/ana.410400518 8957023

[10] Deb S, Braganza J, Norton N, . APOE epsilon 4 influences the manifestation of Alzheimer’s disease in adults with Down’s syndrome. Br J Psychiatry. 2000;176:468-472. doi:10.1192/bjp.176.5.468 10912224

[11] Coppus AM, Evenhuis HM, Verberne GJ, . The impact of apolipoprotein E on dementia in persons with Down’s syndrome. Neurobiol Aging. 2008;29(6):828-835. doi:10.1016/j.neurobiolaging.2006.12.013 17250929

[12] Hyman BT, West HL, Rebeck GW, . Quantitative analysis of senile plaques in Alzheimer disease: observation of log-normal size distribution and molecular epidemiology of differences associated with apolipoprotein E genotype and trisomy 21 (Down syndrome). Proc Natl Acad Sci U S A. 1995;92(8):3586-3590. doi:10.1073/pnas.92.8.3586 7724603 PMC42212

[13] Jack CR Jr, Bennett DA, Blennow K, . A/T/N: an unbiased descriptive classification scheme for Alzheimer disease biomarkers. Neurology. 2016;87(5):539-547. doi:10.1212/WNL.0000000000002923 27371494 PMC4970664

[14] Annus T, Wilson LR, Hong YT, . The pattern of amyloid accumulation in the brains of adults with Down syndrome. Alzheimers Dement. 2016;12(5):538-545. doi:10.1016/j.jalz.2015.07.490 26362596 PMC4867786

[15] Esteba-Castillo S, Dalmau-Bueno A, Ribas-Vidal N, Vilà-Alsina M, Novell-Alsina R, García-Alba J. Adaptation and validation of CAMDEX-DS (Cambridge Examination for Mental Disorders of Older People with Down’s Syndrome and others with intellectual disabilities) in Spanish population with intellectual disabilities [in Spanish]. Rev Neurol. 2013;57(8):337-346. 24081888

[16] Benejam B, Fortea J, Molina-López R, Videla S. Patterns of performance on the modified Cued Recall Test in Spanish adults with Down syndrome with and without dementia. Am J Intellect Dev Disabil. 2015;120(6):481-489. doi:10.1352/1944-7558-120.6.481 26505869

[17] Benejam B, Videla L, Vilaplana E, . Diagnosis of prodromal and Alzheimer’s disease dementia in adults with Down syndrome using neuropsychological tests. Alzheimers Dement (Amst). 2020;12(1):e12047. doi:10.1002/dad2.12047 32613076 PMC7322242

[18] Fortea J, Carmona-Iragui M, Benejam B, . Plasma and CSF biomarkers for the diagnosis of Alzheimer’s disease in adults with Down syndrome: a cross-sectional study. Lancet Neurol. 2018;17(10):860-869. doi:10.1016/S1474-4422(18)30285-0 30172624

[19] Carmona-Iragui M, Santos T, Videla S, . Feasibility of lumbar puncture in the study of cerebrospinal fluid biomarkers for Alzheimer’s disease in subjects with Down syndrome. J Alzheimers Dis. 2017;55(4):1489-1496. doi:10.3233/JAD-160827 27858714

[20] Karikari TK, Pascoal TA, Ashton NJ, . Blood phosphorylated tau 181 as a biomarker for Alzheimer’s disease: a diagnostic performance and prediction modelling study using data from four prospective cohorts. Lancet Neurol. 2020;19(5):422-433. doi:10.1016/S1474-4422(20)30071-5 32333900

[21] Alcolea D, Clarimón J, Carmona-Iragui M, . The Sant Pau Initiative on Neurodegeneration (SPIN) cohort: a data set for biomarker discovery and validation in neurodegenerative disorders. Alzheimers Dement (N Y). 2019;5:597-609. doi:10.1016/j.trci.2019.09.005 31650016 PMC6804606

[22] Landau SM, Mintun MA, Joshi AD, ; Alzheimer’s Disease Neuroimaging Initiative. Amyloid deposition, hypometabolism, and longitudinal cognitive decline. Ann Neurol. 2012;72(4):578-586. doi:10.1002/ana.23650 23109153 PMC3786871

[23] Landau SM, Harvey D, Madison CM, ; Alzheimer’s Disease Neuroimaging Initiative. Associations between cognitive, functional, and FDG-PET measures of decline in AD and MCI. Neurobiol Aging. 2011;32(7):1207-1218. doi:10.1016/j.neurobiolaging.2009.07.002 19660834 PMC2891865

[24] Avants BB, Tustison NJ, Song G, Cook PA, Klein A, Gee JC. A reproducible evaluation of ANTs similarity metric performance in brain image registration. Neuroimage. 2011;54(3):2033-2044. doi:10.1016/j.neuroimage.2010.09.025 20851191 PMC3065962

[25] Handen BL, Cohen AD, Channamalappa U, . Imaging brain amyloid in nondemented young adults with Down syndrome using Pittsburgh compound B. Alzheimers Dement. 2012;8(6):496-501. doi:10.1016/j.jalz.2011.09.229 23102120 PMC3532743

[26] Zammit MD, Laymon CM, Betthauser TJ, . Amyloid accumulation in Down syndrome measured with amyloid load. Alzheimers Dement (Amst). 2020;12(1):e12020. doi:10.1002/dad2.12020 32435686 PMC7233422

[27] Klunk WE, Koeppe RA, Price JC, . The Centiloid Project: standardizing quantitative amyloid plaque estimation by PET. Alzheimers Dement. 2015;11(1):1-15.e1, 4. doi:10.1016/j.jalz.2014.07.003 25443857 PMC4300247

[28] Pastor P, Roe CM, Villegas A, . Apolipoprotein epsilon4 modifies Alzheimer’s disease onset in an E280A PS1 kindred. Ann Neurol. 2003;54(2):163-169. doi:10.1002/ana.10636 12891668

[29] Firth NC, Startin CM, Hithersay R, ; LonDownS Consortium. Aging related cognitive changes associated with Alzheimer’s disease in Down syndrome. Ann Clin Transl Neurol. 2018;5(6):741-751. doi:10.1002/acn3.571 29928657 PMC5989753

[30] Lai F, Kammann E, Rebeck GW, Anderson A, Chen Y, Nixon RA. APOE genotype and gender effects on Alzheimer disease in 100 adults with Down syndrome. Neurology. 1999;53(2):331-336. doi:10.1212/WNL.53.2.331 10430422

[31] Vemuri P, Wiste HJ, Weigand SD, ; Alzheimer’s Disease Neuroimaging Initiative. Effect of apolipoprotein E on biomarkers of amyloid load and neuronal pathology in Alzheimer disease. Ann Neurol. 2010;67(3):308-316. doi:10.1002/ana.21953 20373342 PMC2886799

[32] Baek MS, Cho H, Lee HS, Lee JH, Ryu YH, Lyoo CH. Effect of APOE ε4 genotype on amyloid-β and tau accumulation in Alzheimer’s disease. Alzheimers Res Ther. 2020;12(1):140. doi:10.1186/s13195-020-00710-6 33129364 PMC7603688

[33] Jansen WJ, Ossenkoppele R, Knol DL, ; Amyloid Biomarker Study Group. Prevalence of cerebral amyloid pathology in persons without dementia: a meta-analysis. JAMA. 2015;313(19):1924-1938. doi:10.1001/jama.2015.4668 25988462 PMC4486209

[34] Oxtoby NP, Young AL, Cash DM, . Data-driven models of dominantly-inherited Alzheimer’s disease progression. Brain. 2018;141(5):1529-1544. doi:10.1093/brain/awy050 29579160 PMC5920320

[35] Lim YY, Hassenstab J, Cruchaga C, ; Dominantly Inherited Alzheimer Network. BDNF Val66Met moderates memory impairment, hippocampal function and tau in preclinical autosomal dominant Alzheimer’s disease. Brain. 2016;139(pt 10):2766-2777. doi:10.1093/brain/aww200 27521573 PMC5815565

[36] Henson RL, Doran E, Christian BT, . Cerebrospinal fluid biomarkers of Alzheimer’s disease in a cohort of adults with Down syndrome. Alzheimers Dement (Amst). 2020;12(1):e12057. doi:10.1002/dad2.12057 32671183 PMC7346867

[37] Lemere CA, Blusztajn JK, Yamaguchi H, Wisniewski T, Saido TC, Selkoe DJ. Sequence of deposition of heterogeneous amyloid beta-peptides and APO E in Down syndrome: implications for initial events in amyloid plaque formation. Neurobiol Dis. 1996;3(1):16-32. doi:10.1006/nbdi.1996.0003 9173910

[38] Buckley RF, Mormino EC, Chhatwal J, ; Alzheimer’s Disease Neuroimaging Initiative. Associations between baseline amyloid, sex, and APOE on subsequent tau accumulation in cerebrospinal fluid. Neurobiol Aging. 2019;78:178-185. doi:10.1016/j.neurobiolaging.2019.02.019 30947113 PMC6545139

[39] Hohman TJ, Dumitrescu L, Barnes LL, ; Alzheimer’s Disease Genetics Consortium and the Alzheimer’s Disease Neuroimaging Initiative. Sex-specific association of apolipoprotein E with cerebrospinal fluid levels of tau. JAMA Neurol. 2018;75(8):989-998. doi:10.1001/jamaneurol.2018.0821 29801024 PMC6142927

[40] La Joie R, Visani AV, Lesman-Segev OH, . Association of APOE4 and clinical variability in Alzheimer disease with the pattern of tau- and amyloid-PET. Neurology. 2021;96(5):e650-e661. doi:10.1212/WNL.0000000000011270 33262228 PMC7884991

[41] Emrani S, Arain HA, DeMarshall C, Nuriel T. APOE4 is associated with cognitive and pathological heterogeneity in patients with Alzheimer’s disease: a systematic review. Alzheimers Res Ther. 2020;12(1):141. doi:10.1186/s13195-020-00712-4 33148345 PMC7643479

[42] El Haj M, Antoine P, Amouyel P, . Apolipoprotein E (APOE) ε4 and episodic memory decline in Alzheimer’s disease: a review. Ageing Res Rev. 2016;27:15-22. doi:10.1016/j.arr.2016.02.002 26876367 PMC5114144

[43] Strydom A, Coppus A, Blesa R, . Alzheimer’s disease in Down syndrome: an overlooked population for prevention trials. Alzheimers Dement (N Y). 2018;4:703-713. doi:10.1016/j.trci.2018.10.006 30581976 PMC6296162

[44] Cohen AD, McDade E, Christian B, . Early striatal amyloid deposition distinguishes Down syndrome and autosomal dominant Alzheimer’s disease from late-onset amyloid deposition. Alzheimers Dement. 2018;14(6):743-750. doi:10.1016/j.jalz.2018.01.002 29477284 PMC5994364

[45] Shinohara M, Fujioka S, Murray ME, . Regional distribution of synaptic markers and APP correlate with distinct clinicopathological features in sporadic and familial Alzheimer’s disease. Brain. 2014;137(pt 5):1533-1549. doi:10.1093/brain/awu046 24625695 PMC3999719

[46] Namba Y, Tomonaga M, Kawasaki H, Otomo E, Ikeda K. Apolipoprotein E immunoreactivity in cerebral amyloid deposits and neurofibrillary tangles in Alzheimer’s disease and kuru plaque amyloid in Creutzfeldt-Jakob disease. Brain Res. 1991;541(1):163-166. doi:10.1016/0006-8993(91)91092-F 2029618

[47] Therriault J, Benedet AL, Pascoal TA, . Association of apolipoprotein E ε4 with medial temporal tau independent of amyloid-β. JAMA Neurol. 2020;77(4):470-479. doi:10.1001/jamaneurol.2019.4421 31860000 PMC6990684

[48] Day RJ, McCarty KL, Ockerse KE, Head E, Rohn TT. Proteolytic cleavage of apolipoprotein E in the Down syndrome brain. Aging Dis. 2016;7(3):267-277. doi:10.14336/AD.2015.1020 27330841 PMC4898923

