# Supplementary material for: Association of Apolipoprotein E ɛ4 Allele With Clinical and Multimodal Biomarker Changes of Alzheimer Disease in Adults With Down Syndrome
Source: JAMA Neurol. 2021 Jul 6;78(8):937–47. doi: 10.1001/jamaneurol.2021.1893 (PMC8261691; doi:10.1001/jamaneurol.2021.1893)
Supplement: Supplement. — eMethods. Diagnostic Procedure eTable 1. Demographic Data of the Euploid Controls Used as Reference eTable 2. Demographic Data of the Subsamples for Each Biomarker Modality eTable 3. Demographic, Genetic and Clinical Data for Each Recruiting Site eFigure 1. Effect of APOE ɛ4 on the Age-Related Cognitive Performance in Adults With Down Syndrome With Mild and Moderate Intellectual Disability eFigure 2. Effect of APOE ɛ4 on the Evolution of Cognitive Performance Analyzed by Decades in Adults With Down Syndrome With Mild and Moderate Intellectual Disability eFigure 3. Effect of APOE ɛ4 on the Evolution of Alzheimer Disease Biomarkers Analyzed by Decades in Adults With Down Syndrome eFigure 4. Effect of APOE ɛ4 on the CSF Levels of Aβ1-42, Aβ1-40, and Total Tau in Adults With Down Syndrome eFigure 5. Results of Voxel-Wise Linear Models Showing Lower Grey Matter Glucose Metabolism in APOE ɛ4 Carriers Compared With Non-Carriers eFigure 6. Results of Voxel-Wise Linear Models Showing Lower Grey Matter Volume in APOE ɛ4 Carriers Compared With Non-Carriers [file jamaneurol-e211893-s001.pdf]

Supplementary Online Content

Bejanin A, Iulita MF, Vilaplana E, et al. Association of apolipoprotein E ε4 allele with clinical and multimodal biomarker changes of Alzheimer disease in adults with Down syndrome. *JAMA Neurol.* Published online July 6, 2021. doi:10.1001/jamaneurol.2021.1893

eMethods. Diagnostic Procedure

eTable 1. Demographic Data of the Euploid Controls Used as Reference
eTable 2. Demographic Data of the Subsamples for Each Biomarker Modality
eTable 3. Demographic, Genetic and Clinical Data for Each Recruiting Site
eFigure 1. Effect of *APOE* ε4 on the Age-Related Cognitive Performance in Adults With Down Syndrome With Mild and Moderate Intellectual Disability
eFigure 2. Effect of *APOE* ε4 on the Evolution of Cognitive Performance Analyzed by Decades in Adults With Down Syndrome With Mild and Moderate Intellectual Disability
eFigure 3. Effect of *APOE* ε4 on the Evolution of Alzheimer's Disease Biomarkers Analyzed by Decades in Adults With Down Syndrome
eFigure 4. Effect of *APOE* ε4 on the CSF Levels of $Aß_{1-42}$, $Aß_{1-40}$, and Total Tau in Adults With Down Syndrome
eFigure 5. Results of Voxel-Wise Linear Models Showing Lower Grey Matter Glucose Metabolism in *APOE* ε4 Carriers Compared With Non-Carriers
eFigure 6. Results of Voxel-Wise Linear Models Showing Lower Grey Matter Volume in *APOE* ε4 Carriers Compared With Non-Carriers

This supplementary material has been provided by the authors to give readers additional information about their work.

© 2021 Bejanin A et al. *JAMA Neurology*.

eMethods. Diagnostic Procedure

The diagnostic classification was performed independently by the neuropsychologists and neurologists/psychiatrists who assessed the participants and was debated during a consensus meeting masked to biomarker data. Functional status to differentiate prodromal Alzheimer disease (AD) and AD dementia was assessed on the basis of anamnesis, the Dementia Questionnaire for Persons with Mental retardation, and the CAMDEX-DS to differentiate decline due to cognitive impairment from preexisting intellectual disability, placing a particular emphasis on establishing change from the individual's best level of functioning.

A diagnosis of “asymptomatic” was given when there was no clinical or neuropsychological suspicion of clinical AD (absence of cognitive impairment beyond the intellectual and developmental disabilities (IDD) or functional decline compared to the previous functioning).

A diagnosis of “prodromal AD” was given when there was suspicion of AD but symptoms did not fulfill criteria for dementia (evidence of cognitive impairment without any functional changes).

A diagnosis of “AD dementia” required evidence of cognitive impairment beyond the IDD that interfered with everyday activities (i.e., presence of a functional decline compared to previous functioning)

Some individuals were classified as “Uncertain” when there was evidence of a cognitive impairment and/or a cognitive decline that could be attributed to a pharmaceutical drug, or psychiatric or other medical conditions.

© 2021 Bejanin A et al. *JAMA Neurology*.

eTable 1. Demographic Data of the Euploid Controls Used as Reference

		Euploid controls (n=158)
**Age (years)**		56.4 [51.0;63.7]
**Sex**		
	Female	97 (61.4%)
	Male	61 (38.6%)
APOE genotype		
	ε2/ε2, ε2/ε3, ε2/ε4, ε3/ε3, ε3/ε4, ε4/ε4 (n)	0, 11, 1, 98, 42, 6

Participants were randomly selected from the SPIN cohort (Sant Pau Initiative on Neurodegeneration).

© 2021 Bejanin A et al. *JAMA Neurology*.

eTable 2. Demographic Data of the Subsamples for Each Biomarker Modality

		APOE ε4 non-carriers	*APOE* ε4 carriers	p value	N
**CAMCOG-DS (N)**		**246**	**70**		
	Age (years)	41.3 [32.6;48.2]	44.0 [31.5;49.3]	0.324	316
	Female (%)	109 (44.3%)	35 (50.0%)	0.479	316
	Center: Barcelona (%)	221 (89.8%)	60 (85.7%)	0.451	316
	Diagnostic: Asymptomatic (%)	194 (80.5%)	44 (63.8%)	0.006	310
	ID: Mild, Moderate (%)	78 (31.7%), 168 (68.3%)	23 (32.9%), 47 (67.1%)	0.885	316
**mCRT Immediate recall (N)**		**209**	**54**		
	Age (years)	41.0 [31.6;47.8]	41.9 [31.0;49.5]	0.690	263
	Female (%)	96 (45.9%)	29 (53.7%)	0.386	263
	Center: Barcelona (%)	209 (100.0%)	54 (100.0%)	.	263
	Diagnostic: Asymptomatic (%)	170 (82.1%)	37 (71.2%)	0.116	259
	ID: Mild, Moderate (%)	64 (30.6%), 145 (69.4%)	21 (38.9%), 33 (61.1%)	0.257	263
**mCRT Delayed recall (N)**		**209**	**53**		
	Age (years)	40.9 [32.0;47.8]	41.8 [31.1;49.8]	0.858	262
	Female (%)	98 (46.9%)	28 (52.8%)	0.536	262
	Center: Barcelona (%)	209 (100.0%)	53 (100.0%)	.	262
	Diagnostic: Asymptomatic (%)	172 (83.1%)	37 (72.5%)	0.128	258
	ID: Mild, Moderate (%)	65 (31.1%), 144 (68.9%)	21 (39.6%), 32 (60.4%)	0.254	262
**CSF A$\beta_{1-42/1-40}$ (N)**		**122**	**34**		
	Age (years)	46.7 [37.7;50.7]	46.4 [41.1;50.6]	0.828	156
	Female (%)	55 (45.1%)	14 (41.2%)	0.833	156
	Center: Barcelona (%)	122 (100.0%)	34 (100.0%)	.	156
	Diagnostic: Asymptomatic (%)	67 (56.3%)	16 (48.5%)	0.548	152
	ID: Mild, Moderate (%)	24 (20.0%), 69 (57.5%)	12 (35.3%), 16 (47.1%)	0.177	154
**CSF NfL (N)**		**109**	**30**		
	Age (years)	45.7 [36.9;50.1]	47.9 [39.4;50.7]	0.361	139
	Female (%)	50 (45.9%)	12 (40.0%)	0.715	139
	Center: Barcelona (%)	109 (100.0%)	30 (100.0%)	.	139
	Diagnostic: Asymptomatic (%)	64 (60.4%)	15 (51.7%)	0.532	135
	ID: Mild, Moderate (%)	22 (20.2%), 61 (56.0%)	11 (36.7%), 14 (46.7%)	0.164	139
**CSF pTau181 (N)**		**124**	**34**		
	Age (years)	46.4 [37.6;50.3]	46.4 [41.1;50.6]	0.655	158
	Female (%)	56 (45.2%)	14 (41.2%)	0.826	158
	Center: Barcelona (%)	124 (100.0%)	34 (100.0%)	.	158
	Diagnostic: Asymptomatic (%)	71 (58.7%)	16 (48.5%)	0.396	154
	ID: Mild, Moderate (%)	24 (19.7%), 70 (57.4%)	12 (35.3%), 16 (47.1%)	0.160	156
**CSF total-Tau (N)**		**124**	**34**		
	Age (years)	46.5 [37.6;50.3]	46.4 [41.1;50.6]	0.668	158
	Female (%)	55 (44.4%)	14 (41.2%)	0.892	158
	Center: Barcelona (%)	124 (100.0%)	34 (100.0%)	.	158

© 2021 Bejanin A et al. *JAMA Neurology*.

	Diagnostic: Asymptomatic (%)	71 (58.7%)	16 (48.5%)	0.396	154
	ID: Mild, Moderate (%)	24 (19.7%), 70 (57.4%)	12 (35.3%), 16 (47.1%)	0.160	156
**Plasma NfL (N)**		**277**	**77**		
	Age (years)	43.1 [35.0;49.9]	45.9 [37.3;50.8]	0.221	354
	Female (%)	128 (46.2%)	37 (48.1%)	0.875	354
	Center: Barcelona (%)	277 (100.0%)	77 (100.0%)	.	354
	Diagnostic: Asymptomatic (%)	190 (70.9%)	47 (62.7%)	0.222	343
	ID: Mild, Moderate (%)	58 (21.0%), 149 (54.0%)	20 (26.0%), 38 (49.4%)	0.632	353
**Plasma pTau181 (N)**		**274**	**80**		
	Age (years)	42.9 [33.9;49.8]	46.9 [37.1;50.9]	0.081	354
	Female (%)	130 (47.4%)	39 (48.8%)	0.938	354
	Center: Barcelona (%)	274 (100.0%)	80 (100.0%)	.	354
	Diagnostic: Asymptomatic (%)	192 (72.7%)	50 (64.1%)	0.184	342
	ID: Mild, Moderate (%)	60 (21.9%), 144 (52.6%)	20 (25.0%), 37 (46.2%)	0.611	354
**Amyloid-PET (N)**		**56**	**19**		
	Age (years)	40.5 [33.2;48.7]	43.1 [32.0;49.0]	0.985	75
	Female (%)	23 (41.1%)	5 (26.3%)	0.382	75
	Center: Barcelona (%)	35 (62.5%)	10 (52.6%)	0.626	75
	Diagnostic: Asymptomatic (%)	42 (79.2%)	12 (63.2%)	0.218	72
	ID: Mild, Moderate (%)	20 (35.7%), 33 (58.9%)	6 (31.6%), 10 (52.6%)	0.397	75
**FDG-PET (N)**		**107**	**25**		
	Age (years)	47.2 [38.7;52.8]	44.4 [37.9;50.2]	0.384	132
	Female (%)	50 (46.7%)	9 (36.0%)	0.454	132
	Center: Barcelona (%)	107 (100.0%)	25 (100.0%)	.	132
	Diagnostic: Asymptomatic (%)	60 (58.8%)	13 (52.0%)	0.694	127
	ID: Mild, Moderate (%)	26 (24.3%), 59 (55.1%)	9 (36.0%), 11 (44.0%)	0.467	132
**3T-MRI (N)**		**132**	**43**		
	Age (years)	42.5 [35.1;49.7]	44.5 [31.4;48.9]	0.634	175
	Female (%)	54 (40.9%)	16 (37.2%)	0.802	175
	Center: Barcelona (%)	110 (83.3%)	33 (76.7%)	0.457	175
	Diagnostic: Asymptomatic (%)	96 (74.4%)	28 (65.1%)	0.326	172
	ID: Mild, Moderate (%)	39 (29.8%), 74 (56.5%)	12 (27.9%), 26 (60.5%)	0.888	174

Center = Recruiting center. ID = Intellectual disability. Unless otherwise indicated, values are n (%) or median [Quartile 1; Quartile 3]. Level percentages for intellectual disability and diagnostic group were calculated according to the total of patients with available data in each group. P values refer to analyses of chi-squared tests for categorical variables and Mann-Whitney tests for continuous variables.

© 2021 Bejanin A et al. *JAMA Neurology*.

eTable 3. Demographic, Genetic and Clinical Data for Each Recruiting Site

		Barcelona (N=428)	Cambridge (N=36)	p value	N
**Age (years)**		44.3 [35.0;50.5]	42.2 [34.1;48.6]	0.467	464
**Sex**				0.701	464
	Female	199 (46.5%)	15 (41.7%)		
	Male	229 (53.5%)	21 (58.3%)		
**APOE**				0.161	464
	ε2/ε2, ε2/ε3, ε2/ε4, ε3/ε3, ε3/ε4, ε4/ε4 (n)	1,40,6,300,77,4	0,7,1,19,9,0		
**APOE ε4 status**				0.4	464
	Non-Carriers	341 (79.7%)	26 (72.2%)		
	Carriers	87 (20.3%)	10 (27.8%)		
**Symptoms (%)**				0.321	453
	Asymptomatic AD	282 (67.5%)	27 (77.1%)		
	Symptomatic AD	136 (32.5%)	8 (22.9%)		
**Diagnostic (%)**				0.325	464
	Asymptomatic	282 (65.9%)	27 (75.0%)		
	Prodromal	51 (11.9%)	5 (13.9%)		
	Demented	85 (19.9%)	3 (8.3%)		
	Else/Uncertain	10 (2.3%)	1 (2.8%)		
**Level of intellectual disability (%)**				<0.001	461
	Mild	90 (21.1%)	15 (42.9%)		
	Moderate	220 (51.6%)	20 (57.1%)		
	Severe or profound	116 (27.2%)	0 (0.0%)		
**Cognition**					
	CAMCOG-DS score	70.0 [53.0;82.0]	80.0 [66.0;90.0]	0.004	349
	CAMCOG-DS score (Mild/Moderate ID)	73.0 [59.0;84.0]	80.0 [66.0;90.0]	0.053	348

Unless otherwise indicated, values are n (%) or median [Quartile 1; Quartile 3]. Level percentages for intellectual disability (ID) and diagnostic group were calculated according to the total of patients with available data in each group. P values refer to analyses of chi-squared tests for categorical variables and Mann-Whitney tests for continuous variables. Abbreviations: APOE = apolipoprotein E. CAMCOG-DS=Cambridge Cognitive Examination for Older Adults with Down syndrome.

© 2021 Bejanin A et al. *JAMA Neurology*.

eTable 3 shows that there were no significant differences between the recruiting sites for demographics (age or sex), *APOE* gene frequency (27.8% vs 20.3% individuals carrying the *APOE* ε4 allele) or the disease stage on the AD continuum. The two cohorts only differed for the level of intellectual disability: unlike the cohort of Barcelona, the Cambridge cohort did not include any individual with severe or profound intellectual disability. This is explained by the different sources of recruitment. The cohort of Barcelona is recruited from a population-based health plan developed for the screening of AD in Down syndrome. By contrast, the Cambridge data comes from a convenience sample recruited via services for people with intellectual disabilities in England and Scotland.

It is worth mentioning that this between-cohort difference is unlikely to have influenced our results. Indeed, the Cambridge cohort had a relatively small sample size (n=36) compared to the Barcelona cohort (n=428) and had available data only for a subset of analyses. Moreover, when analyses were repeated excluding the Cambridge data, results remained highly similar (i.e., effect with the same direction and similar strength) even though some specific differences did not reach the statistical threshold due to the smaller sample size.

© 2021 Bejanin A et al. *JAMA Neurology*.

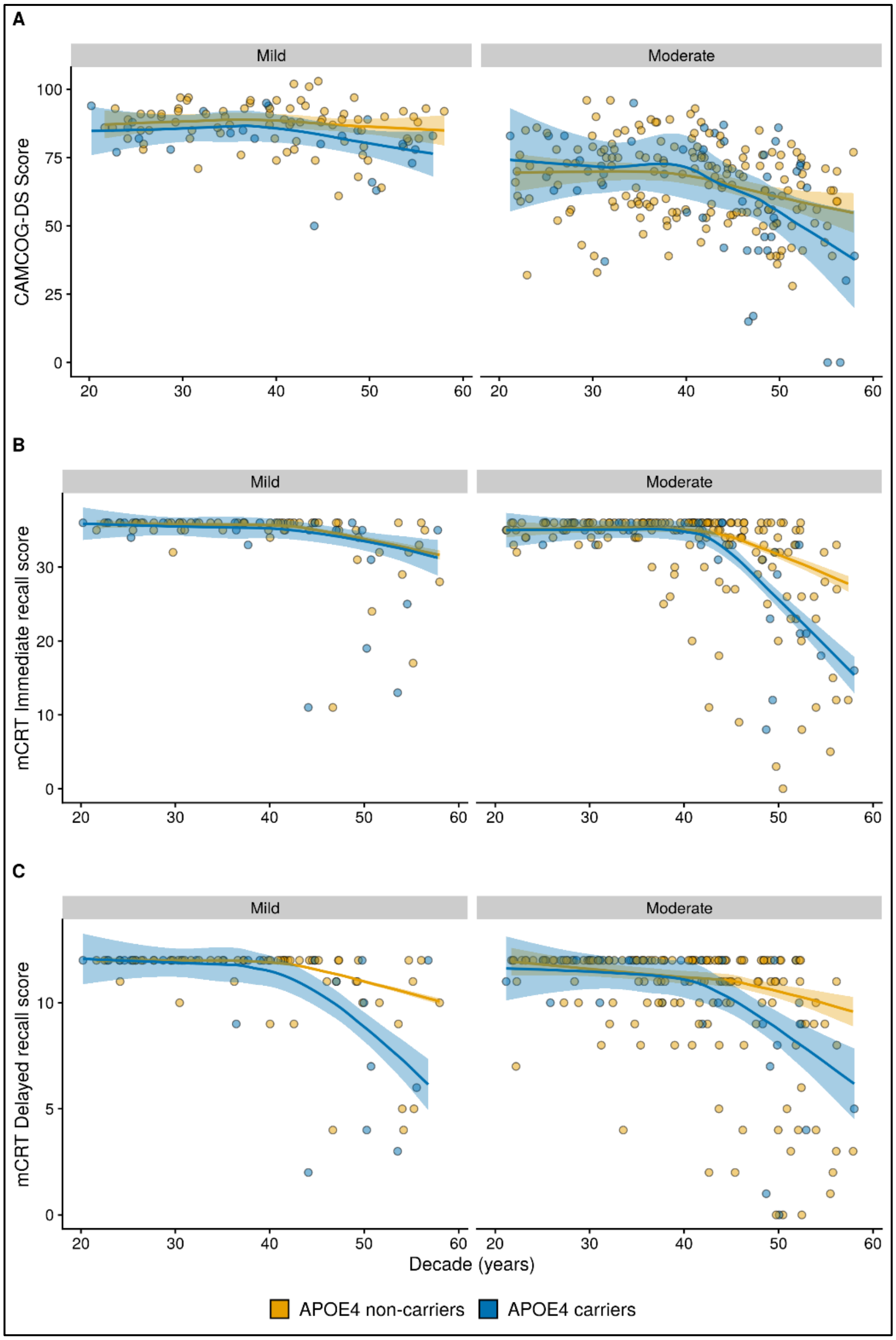

eFigure 1. Effect of *APOE* ε4 on the Age-Related Cognitive Performance in Adults With Down Syndrome With Mild and Moderate Intellectual Disability

Scatterplots showing the age-related changes in performances at the CAMCOG-DS (A), immediate (B), and delayed (C) recall at the mCRT, with bands representing the 95% confidence intervals. CAMCOG-DS = Cambridge Cognitive Examination for Older Adults with Down Syndrome. mCRT = modified Cued Recall Test.

© 2021 Bejanin A et al. *JAMA Neurology*.

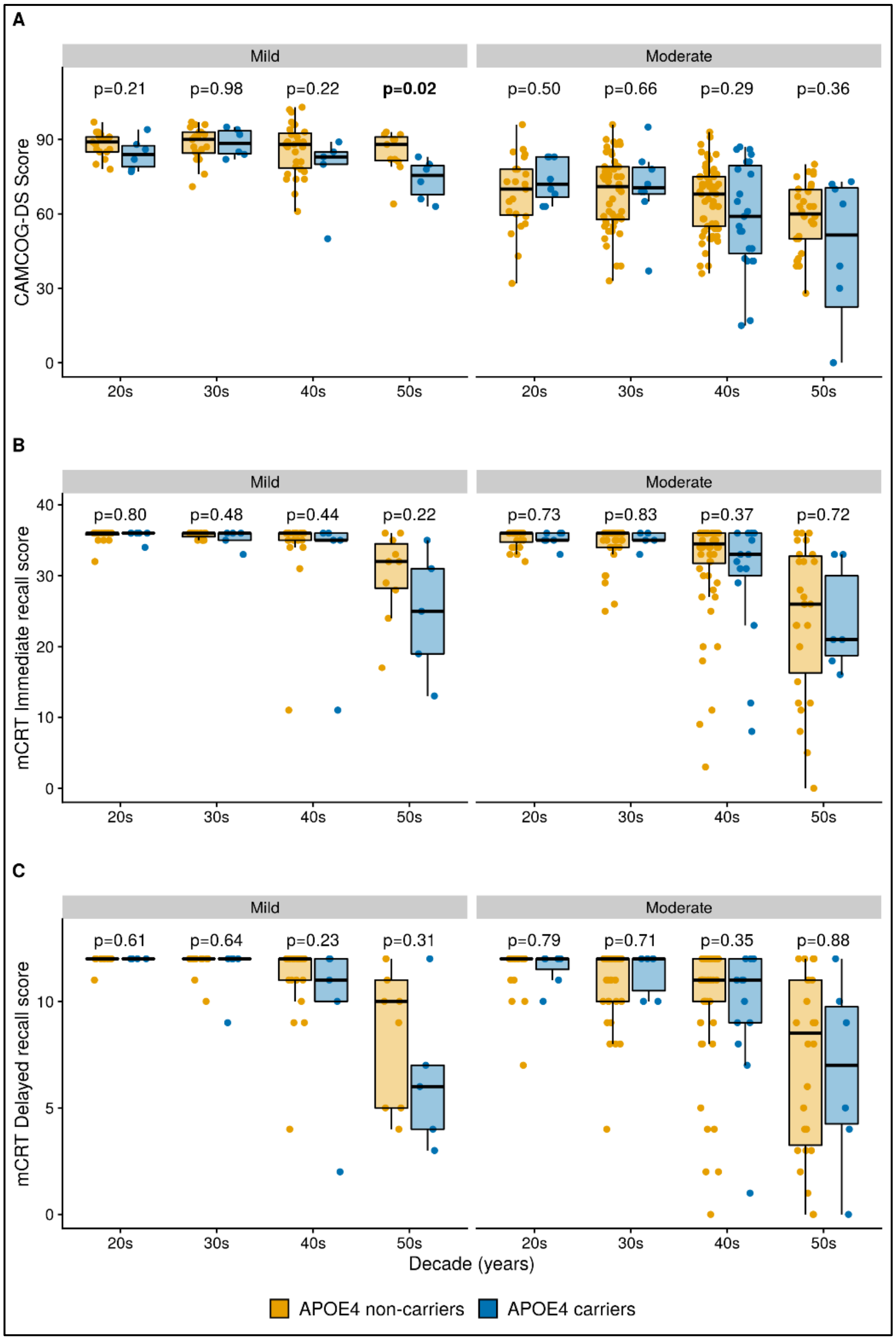

eFigure 2. Effect of *APOE* ε4 on the Evolution of Cognitive Performance Analyzed by Decades in Adults With Down Syndrome With Mild and Moderate Intellectual Disability

Boxplots showing the changes by decades in performances at the CAMCOG-DS (A), immediate (B), and delayed (C) recall at the mCRT. P values refer to analyses of Mann-Whitney test. The bold font indicates statistical significance ($p<0.05$). CAMCOG-DS = Cambridge Cognitive Examination for Older Adults with Down Syndrome. mCRT = modified Cued Recall Test.

© 2021 Bejanin A et al. *JAMA Neurology*.

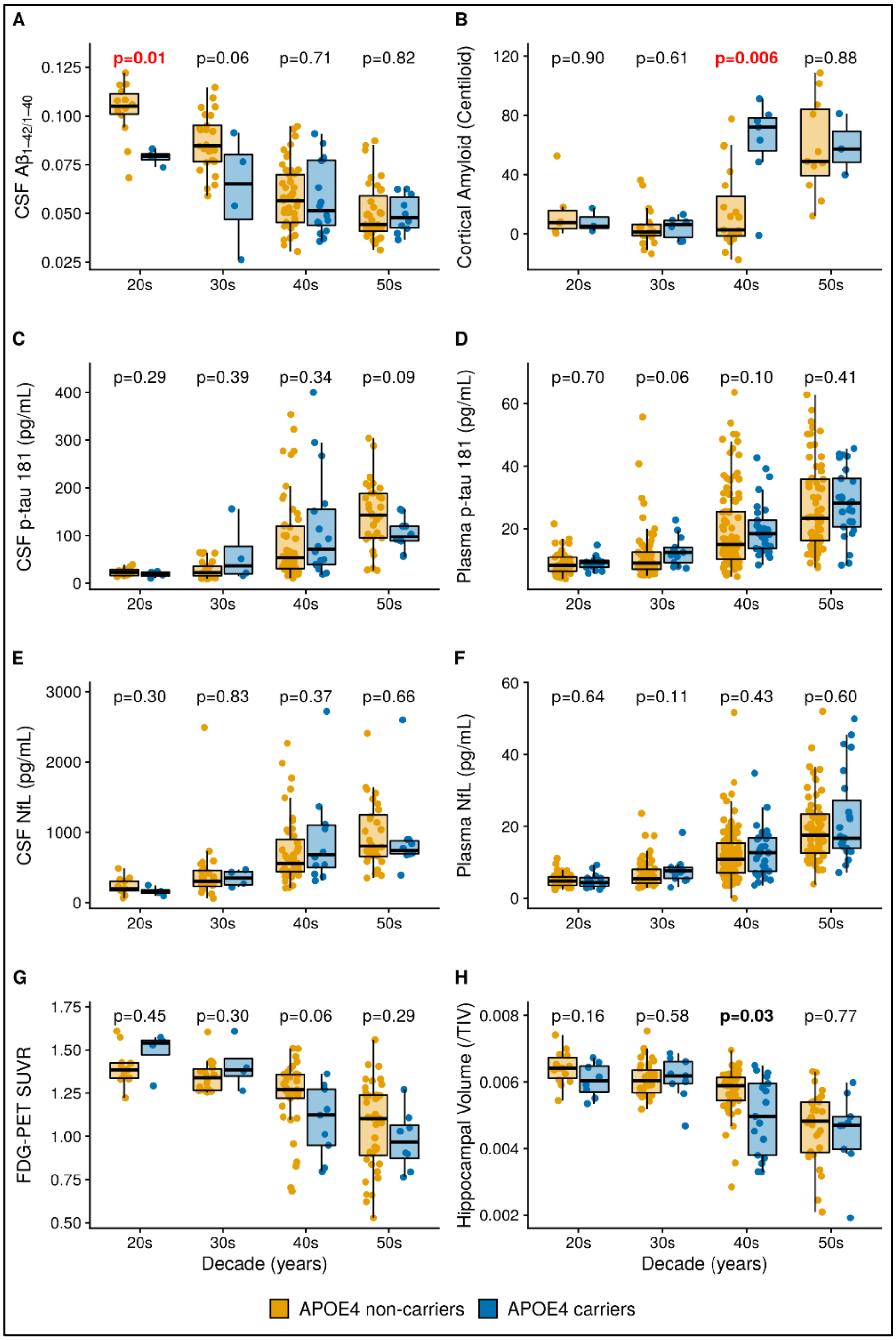

eFigure 3. Effect of *APOE* ε4 on the Evolution of Alzheimer's Disease Biomarkers Analyzed by Decades in Adults With Down Syndrome

Boxplots showing the changes by decades in Alzheimer disease biomarkers in *APOE* ε4 carriers and non-carriers with Down syndrome. P values refer to analyses of Mann-Whitney test. The bold font indicates statistical significance ($p<0.05$), and the red font indicates results surviving the correction for multiple comparisons. Aß$_{1\text{-}40}$=amyloid β peptide 1–40. Aß$_{1\text{-}42}$=amyloid β peptide 1–42. CSF=cerebrospinal fluid. FDG=^{18}F-fluorodeoxyglucose. NfL=neurofilament light chain. SUVR=standardized uptake value ratio. TIV=Total intracranial volume.

© 2021 Bejanin A et al. *JAMA Neurology*.

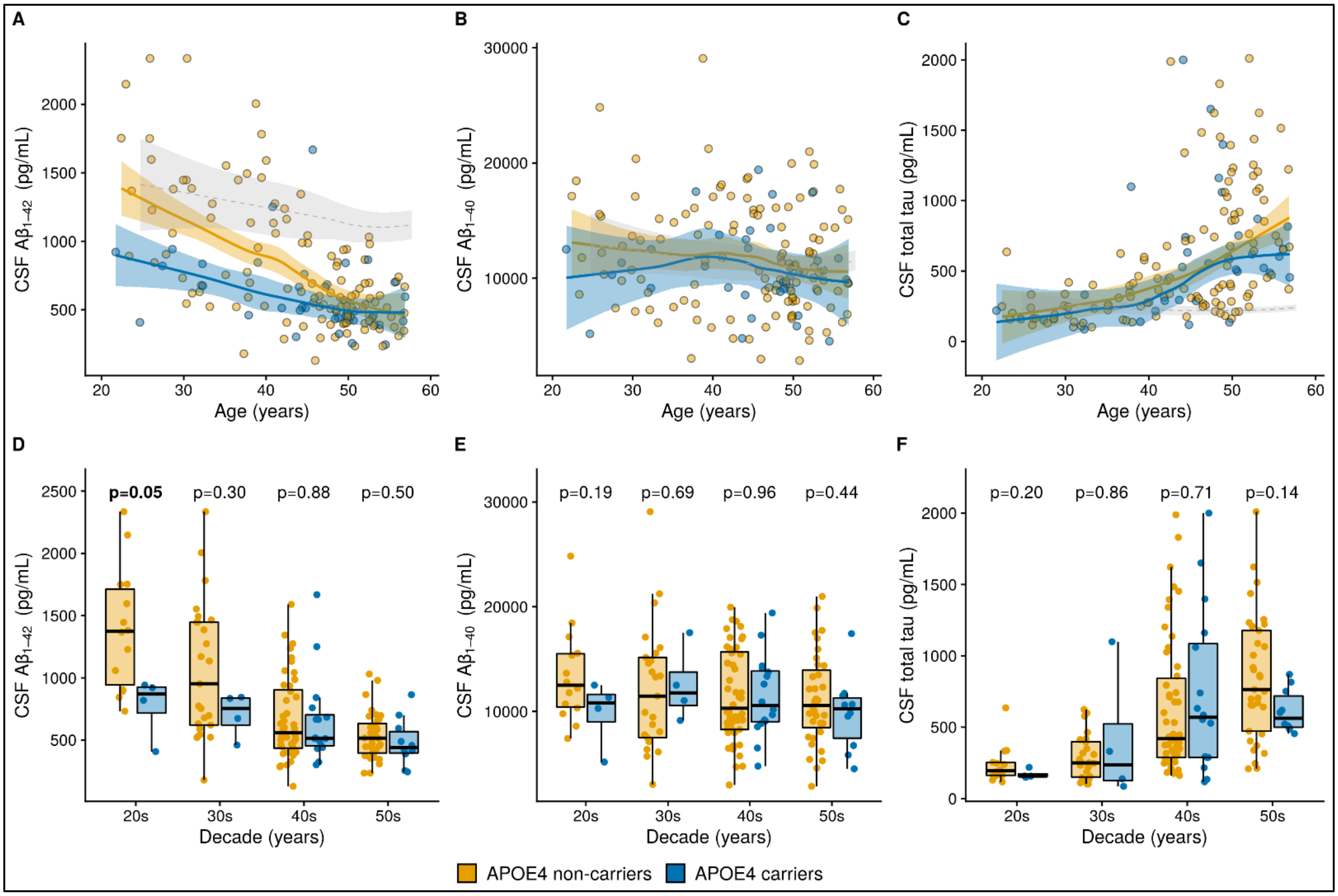

eFigure 4. Effect of *APOE* ε4 on the CSF Levels of Aß1-42, Aß1-40, and Total Tau in Adults With Down Syndrome

Scatterplots and boxplots showing the age-related changes of CSF Aß1-42 (panels A and D), CSF Aß1-40 (panels B and E), and CSF total tau (panels C and F) in *APOE* ε4 carriers and non-carriers with Down syndrome. Shading represents 95% confidence intervals. The grey lines represent the age-related changes in euploid individuals. P values refer to analyses of Mann-Whitney test. The bold font indicates statistical significance ($p<0.05$). Aß1-40=amyloid β peptide 1–40. Aß1-42=amyloid β peptide 1–42. CSF=cerebrospinal fluid.

© 2021 Bejanin A et al. *JAMA Neurology*.

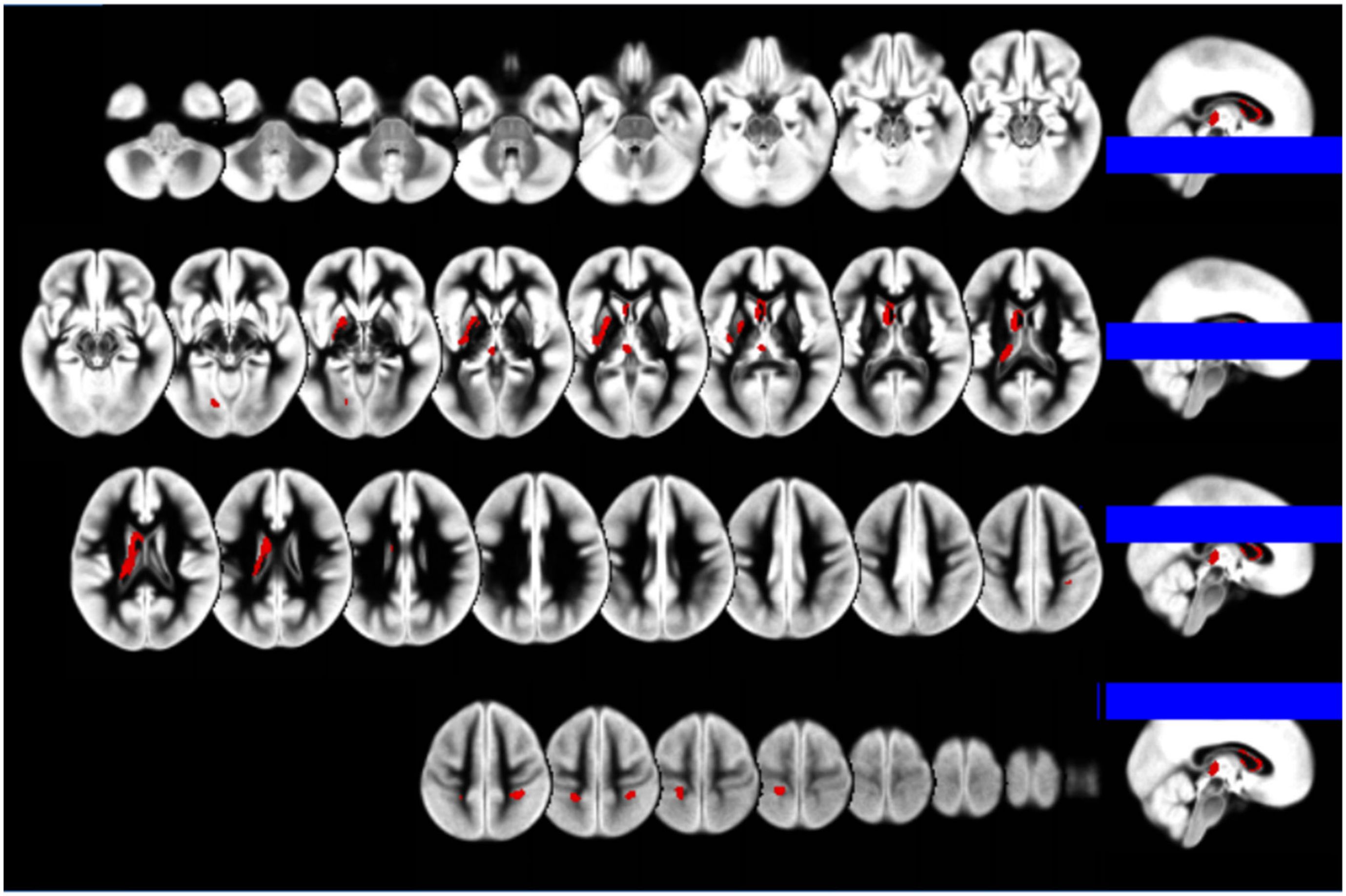

eFigure 5. Results of Voxel-Wise Linear Models Showing Lower Grey Matter Glucose Metabolism in *APOE* ε4 Carriers Compared With Non-Carriers

Results are projected into the SPM template used as reference image for the spatial normalization.

© 2021 Bejanin A et al. *JAMA Neurology*.

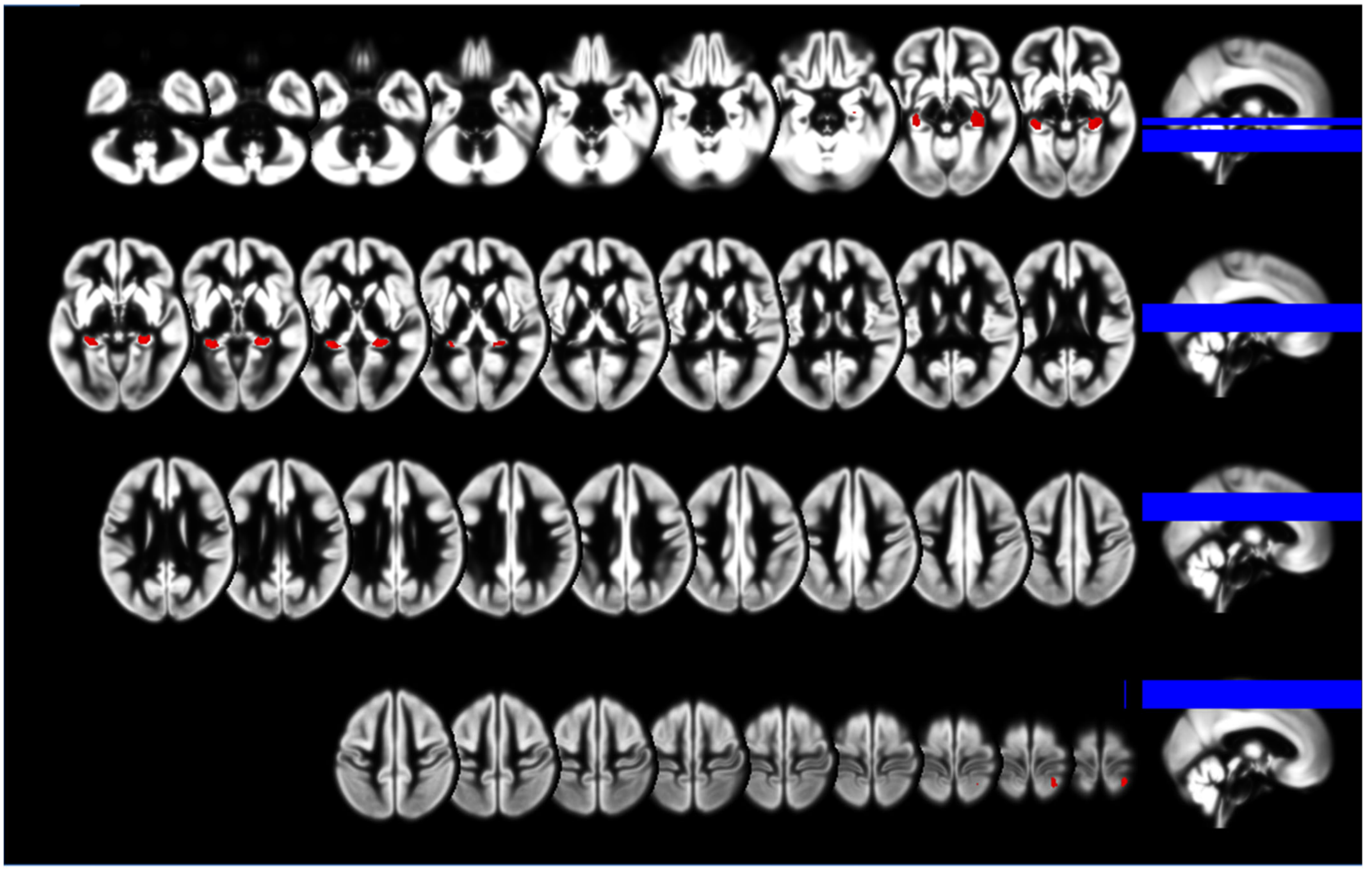

eFigure 6. Results of Voxel-Wise Linear Models Showing Lower Grey Matter Volume in *APOE* ε4 Carriers Compared With Non-Carriers

Results are projected into the SPM template used as reference image for the segmentation and spatial normalization.

© 2021 Bejanin A et al. *JAMA Neurology*.
